# Protocol for a randomized controlled trial comparing wound COmplications in elective midline laparotomies after FAscia Closure using two different Techniques Of Running sutures: COFACTOR trial

**DOI:** 10.1186/s13063-020-04507-8

**Published:** 2020-07-02

**Authors:** Mohamad Hadi El Charif, Zeina Hassan, Jamal Hoballah, Mohamad Khalife, Eman Sbaity

**Affiliations:** Department of Surgery, American University of Beirut Medical Center, Riad El Solh, Beirut, 1107 2020 Lebanon

**Keywords:** Midline laparotomies, Abdominal fascia closure, Wound suture, Incisional hernias, Randomized controlled trial

## Abstract

**Background:**

Wound complications following midline laparotomies are common and the main source of postoperative morbidity including superficial or deep wound infection, skin dehiscence, fascia dehiscence, and incisional hernia. Abdominal closure complications are strongly associated with suture technique and material, in addition to other factors related to the patient and type of surgery performed. The traditional technique is to place the fascia sutures 1 cm apart and at least 1 cm away from the fascia edge. A Swedish study described a new technique of placing the sutures 5 mm apart and 5 mm away from the fascia edge, resulting in lower rates of abdominal wound complications. This study has a number of limitations. There is a need for improved quality evidence to convince the surgical community to change the closure technique of abdominal wounds aiming to reduce morbidity, which is exemplified in incisional hernias and other various postop complications.

**Methods:**

This is a 1:1 randomized, controlled, patient- and assessor-blinded, parallel design, superiority trial, with a primary endpoint of incisional hernia at 1 year. The study will be conducted at AUBMC over a 3-year period. Patients planned for a non-emergent midline laparotomy for general surgery or vascular procedure will be randomized to either fascia closure technique. In order to detect a drop of 12% in the incidence of incisional hernia, with 80% power and an alpha of 0.05, we will need to recruit 114 patients per arm. After adjusting for loss to follow-up, target recruitment is 274 subjects. We will compare both arms for the primary, secondary, and exploratory outcomes, using chi-square or *t* test as appropriate. Univariate and multivariate logistic regression will be done.

**Discussion:**

This trial will assess postop complications following abdominal midline wound closures via two different suturing techniques. This trial will generate evidence-based conclusions that will allow surgeons to assess the role of a new abdominal closure technique in decreasing short- and long-term postoperative complications, for a commonly performed procedure.

**Trial registration:**

ClinicalTrials.gov NCT03527433. Registered on 17 May 2018 before starting participant enrollment.

## Administrative information

Note: the numbers in curly brackets in this protocol refer to SPIRIT checklist item numbers. The order of the items has been modified to group similar items (see http://www.equator-network.org/reporting-guidelines/spirit-2013-statement-defining-standard-protocol-items-for-clinical-trials/).

**Table:** 

Title {1}	**Protocol for A randomized controlled trial comparing wound COmplications in elective midline laparotomies after FAscia Closure using two different Techniques Of Running sutures: COFACTOR-trial.**
Trial registration {2a and 2b}.	ClinicalTrials.gov NCT03527433
Protocol version {3}	Version 7, 25-December-2019
Funding {4}	American University of Beirut Medical Center’s Medical Practice Plan (MPP) Research Fund
Author details {5a}	*Mohamad Hadi El Charif, MD*^*1*^*, Zeina Hassan, MPH*^*1*^*, Jamal Hoballah, MD*^*1*^*, Mohamad Khalife, MD*^*1*^*, Eman Sbaity, MD*^*1*^ ^1^Department of Surgery, American University of Beirut Medical Center
Name and contact information for the trial sponsor {5b}	Eman Sbaity, MD Department of Surgery, AUBMC Riad El Solh, Beirut 1107 2020 Beirut - Lebanon es25@aub.edu.lb
Role of sponsor {5c}	The sponsor is the principal investigator for this study. The sponsor is responsible for the design and setting of the study as well as assigning and supervising the tasks and following up on recruitment, application of interventions, data collection, analysis and results.

## Introduction

### Background and rationale {6a}

Wound complications following midline laparotomies are common and the main source of postoperative morbidity and increased length of hospital stay [1]. Postoperative morbidity includes a spectrum of superficial or deep wound infections, skin dehiscence, and fascia dehiscence with or without evisceration. In addition, incisional hernia is a common delayed postoperative complication occurring in 2–26% of patients undergoing midline laparotomies [2–8]. Fascia dehiscence is a major complication that presents either early with evisceration or late with incisional hernia [3, 8]. Most patients with fascia dehiscence undergo a second surgery for fascia closure, which by itself is associated with morbidity and a high recurrence rate [1].

Abdominal closure complications are strongly associated with suture technique and material, in addition to other factors related to the patient and type of surgery performed. A 2010 survey among surgeons found no consensus with regard to the best technique to close the laparotomy incision [9]. Many RCTs and systematic reviews of the optimal technique to close the abdomen have been reported with heterogeneous results. A recent meta-analysis of 14 trials found significantly lower hernia rates using a continuous versus interrupted suture technique with an odds ratio (OR) of 0.59 (95% confidence interval (CI) 0.43, 0.82) and with slowly absorbable versus rapid absorbable suture material with an OR of 0.65 CI (0.47, 0.9) [10]. Thus, there is adequate evidence for using continuous suture using slowly absorbable material to close the abdominal midline incision. However, the technique of performing continuous suturing is not adequately studied. The traditional way performed by most surgeons is to place the fascia sutures 1 cm apart and at least 1 cm away from the fascia edge.

Several studies in recent years have been conducted to investigate the incisional hernia (IH)-preventive ability of alternative suturing techniques. One trial that is currently recruiting is the HART trial which compares the Hughes Repair to the standard mass closure in patients undergoing colorectal cancer surgery. The study will assess the use of the Hughes Repair as the primary preventive measure for incisional hernias after abdominal surgery [11]. Another trial conducted in 2015 is the STITCH trial, which concluded that small bites sutures (5 mm apart every 5 mm) could reduce the incidence of incisional hernia from 21 to 13%. The STITCH trial was initiated based on the findings of a group of surgeons from Sweden, who described a new technique of placing the sutures closer to each other and closer to the fascial edge [12]. Another Swedish randomized controlled trial was conducted with the aim to have a ratio of 4:1 between the overall length of the suture to the length of the wound being closed. The authors recruited 737 patients, where 381 were allocated to the conventional technique arm with long stitches and 356 were allocated to the new closure technique arm with short stitches. They recruited patients who underwent midline laparotomies for emergency or elective indications. Patients with a previous midline incision, or a pre-existing ventral hernia such as an umbilical or epigastric hernia, were not eligible. Their results showed a lower incidence of wound infections, dehiscence, and incisional hernias with their new fascial closure technique as compared to the conventional one. Outcome measures were defined and assessed clinically. They only assessed fascial dehiscence requiring reoperation [13]. The latter study had a number of limitations discussed further below.

There is a need for better quality evidence to convince the surgical community to implement changes to their closure technique of abdominal wounds. We propose to conduct a randomized controlled trial that addresses the limitations in the design of the existing trials and that will help establish the superiority of the new technique. In this trial we will compare the closure of abdominal wounds, in patients undergoing midline laparotomy incisions, using:

1. Traditional (conventional) closure technique, with the placement of sutures at least 1 cm away from the fascial edge and 1 cm apart from the adjacent fascial suture.
2. Alternative (new) closure technique using smaller and closer fascial sutures, with the placement of sutures only 5 mm away from the fascial edge and 5 mm apart from the adjacent fascial suture.

## Objectives {7}

The primary objective of the COFACTOR study is to determine the relative effects of new versus conventional closure techniques on incisional hernia at 1 year postoperatively in patients undergoing elective midline laparotomies.

The secondary objectives of the trial are to determine the changes in the rates of fascia dehiscence and evisceration within 30 days and the rates of intervention for wound complications in subjects randomized to the new closure technique with short and narrow sutures.

## Trial design {8}

This trial is designed as a 1:1 randomized, controlled, patient- and assessor-blinded, parallel, superiority trial, with the primary endpoint of incisional hernia at 1 year.

In the first 60 days of trial initiation, we will be assessing items related to the institutional systems, the trial itself, and study participants as part of the study's feasibility. We will assess patients' response rate to participate in the trial, acceptance of participants to be examined daily by the research team, adherence of participants to the scheduled postoperative visits, and proportion of participants lost to follow-up. We will also study the compliance of surgeons with conducting the required measurements, the reliability of the randomization and allocation concealment strategy, and the success of blinding of the research team from treatment allocation.

This intensive monitoring period of 60 days will allow us to study the efficacy of the training session and procedure video, consistency of evaluation among different surgeons in performing the outcome assessments, and adequacy of the study team to be available for the scheduled evaluations.

## Methods: participants, interventions, and outcomes

### Study setting {9}

We will conduct the study at the American University of Beirut Medical Center, which is an academic, tertiary referral center.

### Eligibility criteria {10}

#### Surgeons eligibility

General and vascular surgeons who want to enroll their patients in the trial have to be familiar with the new fascia closure technique. They will have to attend a presentation given by the study principal investigator (PI) explaining the details of the new investigational procedure and watch a video demonstrating the new technique during the presentation. At AUBMC, the fascia is typically closed by the surgeon or a senior surgical resident either postgraduate year 4 or 5. Each surgeon or senior resident will be attended to by one of the study team on 3 cases while they close the fascia using the new technique. They will need to successfully demonstrate compliance with the new technique details before they can enroll their patients in the trial.

#### Subject eligibility

##### Inclusion criteria

The following are the inclusion criteria:

1. Age 18 years or older
2. Signed informed consent
3. Undergoing an elective laparotomy through a midline incision
4. Undergoing a midline laparoscopic procedure (midline laparoscopic extraction site of 8 cm or more)

##### Exclusion criteria

The following are the exclusion criteria:

1. Emergency surgery
2. Laparotomy through an incision other than midline
3. Previous midline laparotomy
4. Presence of incisional or ventral hernia at the time of laparotomy
5. Incisional hernia repair
6. Laparotomy surgery during pregnancy
7. Undergoing a midline laparoscopic procedure with a laparoscopic extraction site size of less than 8 cm

### Who will take informed consent? {26a}

After the primary surgeon introduces the study to the patient, the research assistant (RA) will explain the study and invite the patient to sign the consent form. The RA will be trained by the PI on the process of signing the consent. In addition, other members of the study team will be trained and certified to obtain the consent, so they can help in case the RA is not available to discuss participation with a potential candidate. The participant will be given an opportunity to ask questions regarding the study and will receive a copy of the IRB-approved and updated consent form (CF) with his/her signature.

The informed consent is provided in Additional file 1 attached in the submission of this protocol.

### Additional consent provisions for collection and use of participant data and biological specimens {26b}

N/A. The investigators do not expect to conduct ancillary studies requiring the use of participant data that is collected in this study.

## Interventions

### Explanation for the choice of comparators {6b}

The conventional suturing method for abdominal fascia closure is the method of choice for general surgeons at the American University of Beirut Medical Center as well as at international centers. As more trials are released focusing on newer alternative suturing methods, the investigators decided that it is important to assess the complication rates between the conventional and alternative methods in order to modify policies and recommendations for suturing techniques for abdominal fascial closures at their institution.

We theorize that the narrow-suture technique may result in less edema and subsequent swelling to the edges of the wound which allows better healing and less necrosis of the wound edges.

### Intervention description {11a}

The first group will undergo traditional closure with wide and distant sutures, where each suture is placed at least 1 cm away from the fascia edge and 1 cm apart from the adjacent fascia suture.

The second group of participants will undergo the alternative closure with narrow and close fascia sutures, where each suture will be placed only 5 mm away from the fascia edge and 5 mm apart from the adjacent fascia suture.

In the first group, an average of one suture will be placed at each centimeter length of wound; thus, the number of sutures placed should be equal to the length of the wound in centimeters. In the second group, an average of two sutures will be placed at each centimeter length of wound; thus, the number of sutures placed should be equal to at least double the length of the wound in centimeters.

### Criteria for discontinuing or modifying allocated interventions {11b}

The criteria for discontinuing the allocated intervention for the participant will be if he/she requests verbally or in writing to be withdrawn from the trial or indicating preference of one intervention over the other. To note, participants that opt to withdraw from the trial will be included in the intention-to-treat (ITT) analysis.

### Strategies to improve adherence to interventions {11c}

The length of the fascia incision will be measured just before the surgeon starts the closure, and this measurement will be documented by a member of the research team available in the operating room. After the wound is closed, the remaining suture length will be measured by the surgeon and documented as well. For a successful closure of the wound with the new technique, the utilized suture length should be 4 times the length of the wound. Approximately, an additional 10 cm of suture length is needed to ensure proper tying of the knot after the conclusion of the suturing. The remaining suture length should reflect these two considerations. The research team member present in the operative room (OR) will independently calculate the used and remaining suture length. The formula will be as follows: [original length of suture − (length of suture remnants at the starting knot + length of suture remnant at the finishing knot)]/length of the wound. If the final calculation result does not reflect the planned 4:1 ratio between the suture length and wound length, then the participant has to be removed from the study.

If a surgeon fails to ensure the 4:1 closure technique on 3 participants, then these will not be included in the trial and he/she will have to attend the lecture on technique, watch the demonstration videos, and get proctored again on 3 cases, before he/she can resume participating in the trial.

During the duration of hospital stay, it is the responsibility of the PI and the study team to ensure proper and timely assessment of outcome measures, including timely visits by the surgeon evaluator and scheduling of the ultrasound in the radiology department if needed. Upon discharge from the hospital, the participants will be given a calendar as a timeline chart for the remaining follow-up visits' dates. The study team will call the participants at 1 week and at 48 hours before the 30-day appointment and at 2 weeks and at 1 week before the 1-year appointment to ensure that they will show up.

### Relevant concomitant care permitted or prohibited during the trial {11d}

Closure of the subcutaneous tissue and skin will be left at the discretion of the operating surgeon since currently, there is no definitive evidence on the optimal methods, following a laparotomy, for these closures.

Whether to place a regular or closed suction drains or not in the subcutaneous tissue will also be left to the discretion of the operating surgeon, since there is no consensus on this topic in the surgical literature.

Postoperative care of the patient during hospitalization and throughout the duration of the study will be performed according to usual guidelines adopted by each surgeon; thus, this care will vary by surgeon and indication of surgery.

Measures to prevent wound infections will be done following the hospital policy derived from the Centers for Disease Control and Prevention (CDC) guidelines on the prevention of surgical site wound infections. Wound infection will be managed according to the general principles of skin opening and antibiotics.

### Provisions for post-trial care {30}

The adverse effects may be part of the outcome measures detailed later in the protocol or other not specified side effects. Either way, any side effect will be reported, and the participant will be managed according to the standard of care or the preference of the treating surgeon.

### Outcomes {12}

#### Primary outcome measure

- Incisional hernia at 12 months:

We will define an incisional hernia according to the European Society of Hernia: “any abdominal wall gap with or without bulge in the area of a postoperative scar perceptible or palpable by clinical examination or imaging” [14]. Using both clinical exam and imaging, if performed, these hernias will be described according to their location along the midline, size of the defect using vertical and transverse measurements, and reducibility of any protruding viscera upon lying down or following gentle pressure by the examining hand. Also, we will collect information on whether the hernia is causing any pain, discomfort, decrease in mobility, or any incidence of incarceration where the hernia contents protrude and does not reduce to the abdomen upon gentle pressure.

#### Secondary outcome measures

- Fascial dehiscence or evisceration within 30 days:

Fascial dehiscence is defined as the gapping of the fascia by at least 1 cm with a loosening of the surgical sutures. This presents initially with increased serous fluid drainage from the wound. This may be self-limited or progress to a wider gap with herniation of the abdominal viscera, usually the small bowel or the greater omentum, through the defect. The excessive fluid drainage results in the opening of the superficial skin incision in some patients, and this necessitates emergency surgery for repeat closure of the abdominal wall.

- Intervention rate for wound complications within 30 days:

Intervention includes incision and drainage of a wound infection, evacuation of a hematoma, aspiration of a seroma, or reoperation for a wound dehiscence.

#### Exploratory outcomes

1. Wound seroma within 30 days postop:
  - Wound seroma is defined as a collection of serous fluid in the subcutaneous space, detected either clinically or by ultrasound examination.
  - Wound seroma is an established risk factor for wound infection and further resultant morbidities.
2. Wound infection within 30 days postop:

Wound infection will be defined according to the CDC criteria for Surgical Site Infection SSI (see attached Additional file 2 - Appendix A).

Wound infection is a well-known cause of fascial dehiscence. In addition, the fascia closure technique resulting in fascia ischemia will result in deep surgical site infection, which in turn can lead to fascia dehiscence. The Swedish group believes that the wide fascia bite results in a higher risk of fascial ischemia and thus delayed fascial healing or higher risk of deep wound (fascia) infection and higher risk of fascial gapping.

3. Pain during hospitalization:

Pain will be measured using the “0–10 Numeric Pain Rating Scale” [15] (see attached Additional file 2 - Appendix B) on postoperative day (POD) 1, on POD 7, and on discharge (if the participant stays less or more than 7 days). The RA will collect information on pain killers given to the participants postoperatively. This information will be taken into consideration when assessing pain ratings.

4. Quality of life (QOL) at 12 months:

QOL will be measured using the Short Form SF-36 questionnaire (see attached Additional file 2 - Appendix C), which will be validated in the Arabic language [16].

### Participant timeline {13}

#### Enrollment

After the primary surgeon introduces the study to the patient in the outpatient clinics or in the hospital during their admission period before surgery, the RA will approach the patient and discuss the research study with him/her.

#### Assessments and visits

Daily visits to the participants will be conducted by an independent surgeon evaluator or surgical resident while in the hospital for the first 7 days to evaluate relevant outcome measures. The independent surgeon evaluator or surgical resident will examine the wound for possible complications including seroma, hematoma, collections, or fascial gapping. The last evaluation will be on POD 7 or on the day of discharge if the postoperative length of hospital stay is shorter or longer than 7 days.

A similar clinical assessment will be done at 30 days postoperative, with a range of − 7 and + 7 days. Discharged patients will be asked to present to the outpatient clinic for the 30-day assessment visit. Participants remaining hospitalized at 30 days will be visited by the independent surgeon evaluator.

Two or three independent surgeon evaluators will be identified at our center to perform the clinical evaluation. The surgeon evaluator will be either a general surgeon or a vascular surgeon willing to dedicate time for the trial. He/she cannot be the treating surgeon of the study participant. He/she will receive the training session with the remaining faculty who will enroll patients in the trial.

The RA on the study will perform pain assessment on PODs 1 and 7 and at the 30-day postoperative visit.

Twelve months after surgery, the participant will have to come to the outpatient clinic for clinical evaluation of the wound for evidence of incisional hernia and administering the QOL questionnaire. This will be done with a range of ± 30 days. In case the participant cannot reach the clinic due to disability or difficult accessibility, then the independent surgeon evaluator will attempt to visit him/her at their location to perform the clinical assessment of the wound and administer the QOL questionnaire.

### Sample size {14}

Millbourn et al. compared long to short continuous stitches and found a drop in incisional hernia rate at 1 year from 18 to 6% and a drop of SSI from 10 to 5% [6].

In order to detect a drop of 12% in the incidence of incisional hernia, with an alpha of 5%, we will need to recruit 114 patients per arm to detect this difference with an 80% power thus a total of 228 patients need to be recruited into the trial. We are expecting a 30% loss to follow-up, so our adjusted target recruitment will be 274 subjects in total.

After a thorough discussion with our institution’s admitting officers as well as clinicians and surgeons regarding follow-up visit rate, we reached the conclusion that approximately 30% of patients do not present for follow-up by 12 months.

### Recruitment {15}

Possible candidates will be identified from surgery outpatient clinics during the preoperative visit. The treating surgeon will introduce briefly the trial to the patient, and if the patient agrees to participate, he/she will be approached by the RA. If a patient is not approached in the clinic, the RA will approach him/her prior to surgery, only after the treating surgeon gets the patient’s approval.

The expected recruitment rate is 2–3 patients per week. Consequently, it will take 2 years to recruit a total of 274 patients. At AUBMC, we currently perform 300 open laparotomies per 12 months. This recruitment rate will be tested in the first 60 days of the study.

Participants will be reimbursed for transportation and parking fees to cover their trips to our clinics for outcome assessments.

## Assignment of interventions: allocation

### Sequence generation {16a}

Participants will be randomly assigned to their treatment arms in a 1:1 ratio, according to a computer-generated schedule, stratified by type of surgery (vascular or non-vascular), using permuted blocks of variable sizes.

### Concealment mechanism {16b}

Random sequence generation will be selected by the Clinical Research Institute (CRI) biostatistician using a computer software. The CRI biostatistician will hold details of the blocking and block sizes in a separate document unavailable to those involved in the study including those who are enrolling patients, collecting data, evaluating outcomes, or analyzing data, so that we ensure concealment.

The CRI biostatistician will not share the treatment intervention with study personnel until baseline characteristics are collected and the patient is recruited into the trial. The day before the surgery, the surgeon will contact the CRI biostatistician and receive treatment allocation, so he/she can perform the fascia closure technique to which the patient is randomized to.

### Implementation {16c}

The CRI biostatistician, who is not involved in this study, will generate the randomization sequence and keep hold of it. The participants will be recruited and consented by the RA based on eligibility to join the study without any knowledge about their allocation arm. The day before the surgery, the allocation will be revealed by the CRI biostatistician to the treating surgeon to perform the fascia closure. However, none of the study team will be informed about the treatment allocation.

## Assignment of interventions: blinding

### Who will be blinded {17a}

It is impossible to blind the operating surgeon to the allocation, but the rest of the study team will be blinded. We will blind to allocation the independent surgeon evaluator or surgical resident who will assess for outcomes, the RA who is collecting the data, data analysts, the surgical team taking care of the patient (excluding the operating surgeon and residents on the case), and participants themselves. The operating surgeon and residents on the case will be strongly instructed not to disclose allocation status to any of the study team or the patient. The two closure techniques will be referred to and entered in datasheets as A and B, without knowledge of what A and B are.

If wound complications occur that necessitate reoperation for fascia dehiscence, then the re-closure technique will be left to the discretion of the operating surgeon.

### Procedure for unblinding if needed {17b}

If the Data Monitoring Committee (DMC) identifies alerting side effects and the need for unblinding, then that will be done for the DMC members. In addition, if the participant suffers a side effect or complication that the treating surgeon deems as imperative cause for unblinding for better patient care and safety, then intervention allocation to that participant will be revealed and he/she will be removed from the trial.

## Data collection and management

### Plans for assessment and collection of outcomes {18a}

Data on basic demographics of the study participants, relevant risk factors, confounders, and indications for laparotomy surgery will be collected at baseline using protocol-specific standardized case report forms (CRFs). These will be developed to reflect the e-forms that will be developed for the protocol. The CRF will be completed by the RA, following informed consent and prior to the surgical procedure. Data will be collected directly from the participant. The PI will train the RA to properly fill the CRF.

Surgeons involved with the outcome assessment will attend a presentation detailing the definitions of the different outcomes and discuss the standardized measurement procedure. During the first 60 days from the study start day, all independent surgeon evaluators will independently assess the outcomes of the participants and inter-rater reliability will be measured. In case of inconsistency of results, the reasons behind the discrepancy will be investigated and addressed by redefining the outcome measures, clarifying measurement techniques, or further training. Consistency of evaluation among different surgeons will be evaluated periodically, every 2 months.

**Fig. 1:**
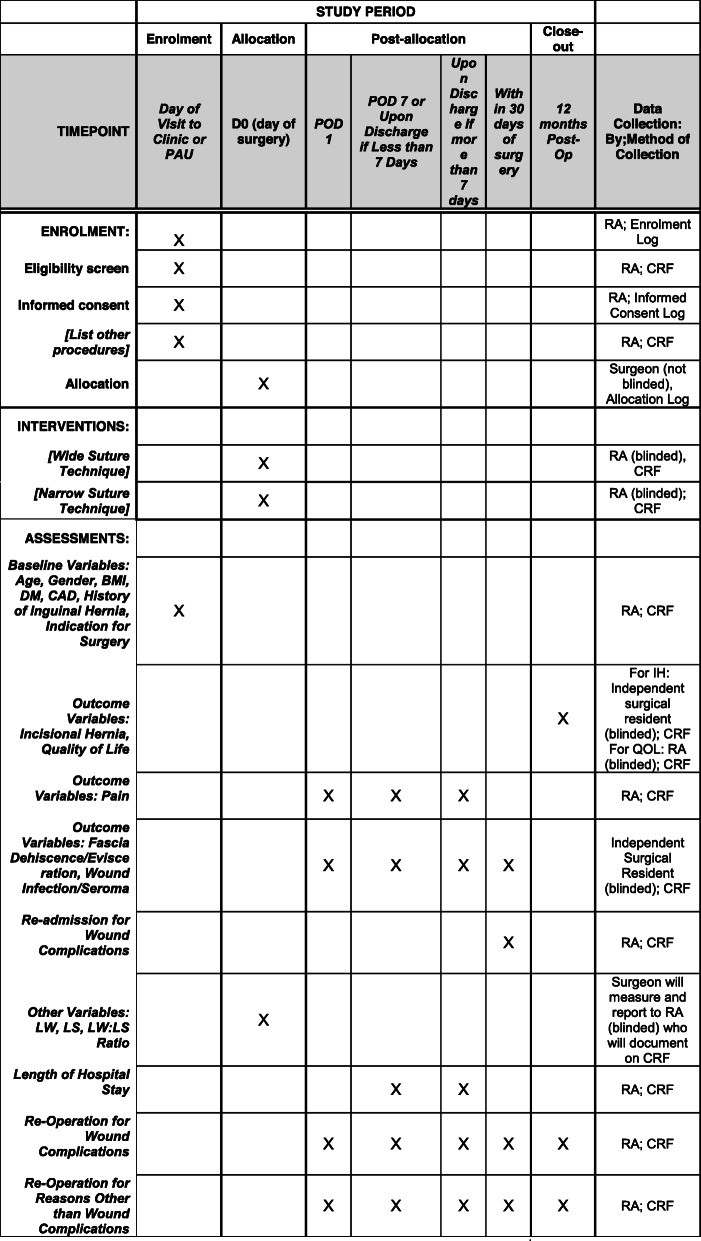
SPIRIT figure for the COFACTOR trial: enrollment, interventions, and assessments

The data on outcomes will be collected at different points of time of the study, as indicated in the SPIRIT figure (Fig. 1), by questionnaires administered by the study's RA or physical examination by an independent surgeon evaluator or surgical resident. The RA will be blinded as well as the independent surgeon evaluator or surgical resident involved. The treating surgeon, who is not blinded, will measure the suture and incision lengths and report them to the RA who will document the information during operation.

Evaluators will be required to fill immediately a CRF each time they will perform an outcome evaluation on a study participant. These forms will be handed in the same setting to the RA who will take care of storing them.

The will evaluate the participant's pain score using the “0–10 Numeric Pain Rating Scale”.

QOL data will be collected using the validated Arabic version of the Short Form SF-35.

### Plans to promote participant retention and complete follow-up {18b}

Participants will be contacted by the research team on bi-monthly basis to assess whether a clinical follow-up is required or not. Participants will be reminded with each communication of their imperative role in the study and the necessity for continuous contact and monitoring till the follow-up period is complete.

At 1 year follow-up (± 30 days) visit, the study team will contact the participant ahead of time and compensate them for transportation, parking, and meals. We will make sure that the allocated periods for research visits are available at different times during the day and on different days of the week, including few slots on Saturdays for those who cannot make it during the week.

When a participant fails to comply with the follow-up visits, we will collect applicable data on the phone. For those who drop out from the trial, we will get their permission to contact them by a phone call at 1 year after their surgery to check whether they developed a clinically detectable incisional hernia and if they have had any intervention for hernia.

### Data management {19}

The RA will enter the data from the CRFs on the study data Excel sheet within 1 week of its collection. A clear explanation of all headings and variables in the data collection sheet will be done on a separate Word document for future reference. Coding of the data will be clarified from the start on a separate sheet within the same document. The data will be manually entered twice and independently by the RA and another member of the study team. Data will be entered as the actual numeric values or the actual categorical variable, initially. In the end, the statistician will code all the data in preparation for analysis. The PI will perform spot checks on the data and will review weekly all the CRFs and assessment log sheets performed within each week.

The PI will plan weekly meetings with the research team to raise and discuss any issues related to data collection, missing data, and retention of patients.

We will have a password-protected laptop for the RA. Moreover, at AUBMC, data will be stored on the AUB intranet server.

The CRFs will be stored in a locked cabinet in the PI’s office for 3 years following the publication of trial results.

As indicated before, the data will be stored on a password-secured laptop, on AUB’s intranet server, and as hard copies in a locked cabinet thus assuring security and backup.

We will formulate a standard operating manual (SOP) detailing data management procedure to ensure consistency in case of a change in the research team members.

Audits will be performed monthly on 20% of the charts.

### Confidentiality {27}

All study-related forms and information will be stored in cabinets that can only be accessed by research team members. All electronic databases will be password protected. Computers used during this trial will also be password protected.

### Plans for collection, laboratory evaluation, and storage of biological specimens for genetic or molecular analysis in this trial/future use {33}

N/A. The investigators will not be collecting or storing any specimens or laboratory values for this trial.

## Statistical methods

### Statistical methods for primary and secondary outcomes {20a}

The intervention arm (short and narrow stitches) will be compared to the standard arm (long and wide stitches) for the primary, secondary, and exploratory outcomes. We will use the chi-square or Fisher exact test if the expected count of any of the outcomes is less than 5 per cell for analysis of the incidence of dichotomous outcomes (fascia dehiscence, incisional hernia, wound seroma, wound infection, and intervention for wound complications). We will use an independent *t* test for the analysis of the continuous outcomes (pain score and QOL measurement). We will calculate relative risk with corresponding 95% confidence intervals to compare the incidence of the dichotomous outcomes, and we will report the difference in means for the continuous outcomes. SPSS version 20 will be used to conduct the analysis. A 2-sided *p* value will be set at 5%.

A univariate analysis will be performed separately for the primary outcome and the two secondary outcomes. The univariate analysis will be conducted separately for incisional hernia at 1 year and intervention for wound complications and wound dehiscence at 30 days postoperative as the dependent variables while vascular indications, diabetes status, BMI, incision length, wound classification (clean, clean-contaminated, or contaminated), and operating surgeon as the independent variables. BMI will be categorized as normal, overweight, obese, and morbidly obese according to consensus BMI value cutoffs for these definitions (see attached Additional file 2). Wound status will be classified according to ACS wound classification system (see attached Additional file 2).

Multivariate analysis using logistic regression will be performed looking at how the primary outcome and each of the secondary outcome measures are affected by each of the above-listed covariates.

We will perform a subgroup analysis for the primary and secondary outcomes using chi-square according to the following variables: operation for obese versus non-obese patients. We anticipate that in obese patients, the seroma and wound infections will be significantly less in the intervention versus the standard group. We also anticipate that the improvement in primary outcomes, especially in the incidence of incisional hernia at 1 year, will be more pronounced in the surgeries for groups not carrying risk factors for IH.

We will perform both ITT and per-protocol (PP) analysis for all outcome measures. The ITT analysis will include all participants in the arm to which they were randomized. Multiple imputation methods will be used to handle missing data. To assess the effect of missing data on the analysis, sensitivity analyses will be performed. The study's biostatistician will perform the best-case scenario, worst-case scenario, and group averages. For outcome measures missing from the 30-day assessment but available at discharge from the hospital, the biostatistician will use the approach of “last observation carried further”. We will assess the baseline characteristics of those who will be lost to follow-up, to help us understand what the potential outcomes were.

### Interim analyses {21b}

The interim analysis will be done 3 times throughout the study; upon recruiting quarter, two-quarters, and three-quarters of the study participants’ population. The study biostatistician who is blind to the treatment allocation will conduct the interim analysis and will use the O’Brien Fleming stopping rules. The study biostatistician will report the results of the interim analysis to the DMC confidentially. At each interim analysis interpretation, the DMC will alert the PI if one arm is found to be beyond doubt either more beneficial or more harmful than the other arm. The PI will take into consideration the results of the interim analysis, opinion of the DMC, and various important factors to decide upon the fate of the trial. The chairperson of the DMC will monitor the ClinicalTrials.gov website for registration of new trials and for newly reported results from trials addressing a similar question as this trial.

### Methods for additional analyses (e.g., subgroup analyses) {20b}

N/A. There are no expected subgroup analyses, other than the ones mentioned in section {20a}, at the time of formulating this protocol.

### Methods in analysis to handle protocol non-adherence and any statistical methods to handle missing data {20c}

We will use ITT analysis, and missing data will be handled using multiple imputations.

### Plans to give access to the full protocol, participant-level data, and statistical code {31c}

The full protocol is currently accessible through ClinicalTrials.gov. Participant-level data and statistical code will be accessible through the methods and results sections published once the trial is concluded and a manuscript is formulated. We are willing to share participant-level data and statistical code after study completion and publication upon reasonable request from the PI.

## Oversight and monitoring

### Composition of the coordinating center and trial steering committee {5d}

Since this trial is a single-center study, then the DMC will be the steering committee which is usually a separate committee required for multi-center studies.

### Composition of the data monitoring committee, its role, and reporting structure {21a}

The DMC committee will be independent of the PI and the funders of the trial. It will be composed of a surgeon, an internist, two nurses, a biostatistician, and a representative from the patient advocacy office at AUBMC. The surgeon will be either a general or vascular surgeon. The internist will have a clinical research background and, if possible, an administration background. The nurses will be chosen from the internal medicine floors or outpatient clinics, to make sure that they will not be taking care of any of the study participants. All members should not have any conflict of interest related to the trial. The DMC will be chaired by the internist, who will have to keep a record of the meetings and recommendations for future reference. Since this is an investigator-initiated trial, the PI will appoint the DMC members.

The DMC will meet every other month, at times of scheduled interim analysis, and upon conclusion of the trial. The primary role of the DMC will be to review the accumulating data and discuss with the PI if there are alarming rates of side effects in any arm of the trial. This committee will not have executive power to stop the trial or modify treatment but can make a recommendation for the former or latter. In addition, the DMC will keep track of the accrual rate.

In the instance where a premature termination of the trial is being considered, the PI will consult with the DMC and other involved personnel from the ethics office to discuss and produce an informed decision. Causes for trial termination can include, but not limited to, the rate and severity of adverse effects and complications as well as inadequate patient recruitment or significant data mishandling that could jeopardize the credibility, accuracy, and reliability of the results.

### Adverse event reporting and harms {22}

The adverse effects can be part of the outcome measures detailed earlier in the protocol or other not specified side effects. Either way, any side effect will be reported, and the participant will be managed according to the standard of care or the preference of the treating surgeon.

### Frequency and plans for auditing trial conduct {23}

The PI will schedule a weekly meeting with all the research team members to review the eligibility of new participants enrolled in the study, consent forms, all CRFs, all assessment log sheets filled during that week, adherence to the trial interventions and policies, and reports of side effects. The PI will double-check the entered data in terms of completeness, timeliness of entry, and correctness of the data. Random checks will also be done.

### Plans for communicating important protocol amendments to relevant parties (e.g., trial participants, ethical committees) {25}

Any modifications to the protocol regarding study objectives, study design, eligibility criteria, sample sizes, or significant changes in the study that will impact study conduct, potential benefit, or safety of the study participants will initially require agreement from the DMC. Then, the amendment will be submitted to the IRB for approval before implementation. The study participants will be notified of study changes and will sign an updated informed consent form reflecting such changes.

## Dissemination plans {31a}

All data and analysis will remain blinded until the main outcomes are published. The trial results will be communicated to the participants by email, letter by mail, or a phone call by the PI.

We will submit a de-identified dataset to an appropriate data archive after 3 years of trial termination to share our data with the surgical community.

Both the trial’s protocol and final findings’ manuscripts will be submitted for publication. Protocol design and trial findings will also be submitted in different forms of presentation at national, regional, and international medical venues addressing relevant issues.

## Discussion

An in-depth search of the literature yields different theories as to the pathophysiological advantage of a narrow-suture technique to the development of an incisional hernia. Hope et al. report that gaps between healing edges allow for scar tissue to fill in and predisposes to incisional hernia [17]. We also theorize that a narrow suturing of the wound can limit edema and swelling of its edges thus allowing for better healing and less necrosis.

In addition to the poorly understood but well-observed advantageous nature of narrow sutures on the prevention of abdominal incisional hernias, studies assessing for this type of suture and comparing it with other suturing techniques also carry a number of limitations, which may limit the generalizability of their results. One such trial is the Swedish study mentioned earlier [13]. The latter study’s allocation method was a pseudo-randomization rather than a true randomization of the study participants. Patients undergoing laparotomies in 1 week were allocated to one treatment arm, and those undergoing laparotomies in the following week were all allocated to the other treatment arm. This is an old and obsolete randomization strategy. Another limitation is the lack of standardization of the suture size: they used 1-0 loop PDS sutures in the conventional closure technique and 2-0 loop PDS sutures in the short and close suturing technique. The authors vaguely described how they did their measurements to ensure proper distances and suture lengths. The study population was not well described where much information about comorbidities and risk factors of dehiscence is not reported. This makes it difficult to comment on generalizability and patient eligibility for the new technique. The authors included both elective and emergency operations which will cause heterogeneity of the results since emergency cases are more prone to develop abdominal wound complications. In addition, the authors did not clarify whether they implemented established guidelines intended to prevent surgical site infection (SSI), where SSI is a major risk factor for failure of abdominal closure and consequent dehiscence or incisional hernia. Non-compliance with these guidelines in their practices would confound the results [13]. Thus, this study provides low-quality evidence. However, despite its limitations, this study presents interesting results and a potential for decreasing complication rates for a commonly performed procedure.

Investigating the prevention of incisional hernias using different suturing techniques requires adequate detection of this complication as a prerequisite. To that effect, the modality for the detection of incisional hernias remains subjective to the experience of the physician or investigator. Some of the more readily accessible modalities are evaluated in the literature and those fall under imaging modalities such as CT scan and Ultrasound or under clinical modalities such as physical examination [18]. Some studies report that CT scan brings accuracy to the detection of ventral abdominal hernias and can be superior to other tools [19–21]. The authors report that CT scan offers sensitivity and specificity ranging between 83 to 100% and 67 to 97%, respectively [19, 20]. However, other studies argue that inter- and intra-observer variability render CT scan as an unreliable means for the detection of ventral abdominal hernias [22]. On the other hand, some studies showed that ultrasound can be a superior modality for incisional hernia detection with a sensitivity ranging between 70 and 98% and a specificity between 88 and 100% [23–25]. Nevertheless, major studies such as the STITCH trial relied on physical examination as the primary means of detecting incisional hernias [12]. In addition, several studies showed that the clinical detection of ventral abdominal incisional hernias is a simple, rapid, radiation-free, and cost-effective method to rely on [26, 27]. It may be argued that imaging modalities bring on the added benefit of abdominal incisional hernia diagnosis in cases that are either asymptomatic or of uncertainty [19, 23]. In our study, we elected to use physical examination as the method for abdominal incisional hernia detection because we are seldom interested in the clinically irrelevant hernias.

The search for alternative suturing techniques that allow for lower rates of postop complications such as incisional hernias has been and will continue to be an interesting medical issue requiring investigation. As such and with the recent introduction of the hereby investigated narrow-suture technique, more research in different settings of populations’ backgrounds, cultures, and socioeconomic statuses would be a valuable addition to the literature on the topic.

The results of this study will allow surgeons to assess the role of a new abdominal closure technique in decreasing short- and long-term postoperative complications, for a commonly performed procedure. This trial is expected to generate evidence-based conclusions that can influence and shape-up future recommendations in the surgical community.

There are no practical or operational issues in this study for the time being. All study-related forms and information will be stored at the study site, where they are stored in cabinets that can only be accessed by study members. All electronic databases will be password protected. Computers used during this study will also be password protected.

## Trial status

NCT03527433 protocol version number 7, 25 December 2019

The recruitment phase began in October 2019, but no patients have been recruited to the date of this protocol submission for publication due to local events and the COVID-19 pandemic. Recruitment phase completion is expected by October 2021.

## Supplementary information

**Additional file 1.** Consent to participate in a research study.

**Additional file 2.** Appendices.

## Data Availability

The data that support the findings of this study will be available from the American University of Beirut Medical Center, but restrictions apply to the availability of these data, which were used under license for the current study, and so are not publicly available. Data are however available from the authors upon reasonable request and with permission of the American University of Beirut Medical Center.

## Abbreviations

QOL: Quality of life
AUBMC: American University of Beirut Medical Center
RCT: Randomized controlled trial
IH: Incisional hernia
HART: Hughes Abdominal Repair Trial
STITCH: Small Bites Versus Large Bites for Closure of Abdominal Midline Incisions Trial
PDS: Polydioxanone suture
SSI: Surgical site infection
CT: Computed tomography
PI: Principal investigator
CDC: Center for Disease Control and Prevention
NRS: Numeric Rating Scale
RA: Research assistant
POD: Postoperation/postop day
SF: Short form
CRI: Clinical Research Institute
CRF: Case report form
SOP: Standard operating manual
SPSS: Statistical Package for Social Sciences
BMI: Body mass index
ACS: American College of Surgeons
ITT: Intention-to-treat
PP: Per-protocol
DMC: Data Monitoring Committee
IRB: Institutional Review Board
CF: Consent form
