# Supplementary material for: Protocol for a randomized controlled trial comparing wound COmplications in elective midline laparotomies after FAscia Closure using two different Techniques Of Running sutures: COFACTOR trial
Source: Trials. 2020 Jul 2;21:608. doi: 10.1186/s13063-020-04507-8 (PMC7330541; doi:10.1186/s13063-020-04507-8)
Supplement: Supplementary file 1 — Additional file 1. Consent to participate in a research study. [file 13063_2020_4507_MOESM1_ESM.doc]

# Consent to Participate in a Research Study

**Title of Research Study:**

**A randomized, controlled trial comparing wound COmplications in elective midline laparotomies after FAscia Closure using two different Techniques Of Running sutures: COFACTOR-trial.**

**Investigator: Dr. Eman Sbaity**

Address: American University Hospital

Cairo Street

Beirut, Lebanon

Phone: (01) 350 000 ext 5260

**Site where the study will be conducted:** American University of Beirut Medical Center, Beirut, Lebanon

**You are being asked to participate in a clinical research study conducted at the American University of Beirut. Please take time to read the following information carefully before you decide whether you want to take part in this study or not. Feel free to ask your doctor if you need more information or clarification about what is stated in this form and the study as a whole.**

**PART I: Information Sheet**

**1) Purpose of the research study and overview of participation:**

Abdominal surgery can be complicated with opening of the deep layers of the wound resulting in longer stay in the hospital and other problems, and may necessitate doing another surgery to close the abdomen. In addition, few months later up to a year, many patients get hernia at the abdominal wound. Surgeons close the abdomen with a similar classic technique. There is a new technique to close the deep abdominal wound and we expect it has better results and prevents opening of the deep abdominal wound and decrease risk of having abdominal hernia later. In the classic technique, surgeons close the abdomen using wide and large stitches; in the new technique, they use small and narrow stitches.

We expect to recruit an approximate of 274 participants, with a total of 137 patients in each arm. Your treating surgeon will approach you and introduce the study during your admission period, before the surgery is performed. The research assistant will further discuss the study with you.

If you agree to participate in this study, you will be assigned by chance to receive the classic or the new abdominal closure technique. You will not know which closure you will receive. If you agree to share in this study, we will collect information about your results at different points in time as follows. We will collect basic information about your health and reason for surgery before you do your surgery. After your surgery, you will be visited daily by a study team member who will check on your wound searching for any signs of infection, fluid collection, or opening of the wound. After one day post-operative, seven days post-operative and on discharge, a member of the research team will measure your pain using the “0-10 Numeric Pain Rating Scale”. The duration of this assessment will take 5 minutes. On the day of discharge, a member of the study team will visit you again and perform another check on your wounds. When you come to see your surgeon after discharge, a member of the research team will check again on your wound. We ask you to come again to the clinic 30 days after your surgery, so the research team can check on your wound. The charges for this extra clinic visit and will be covered by the research money. You will also be compensated for transportation and parking during your 30 day clinic visit. You will be compensated 25$ for the transportation and parking of each visit. The duration of each visit will be of 30 minutes.

We will ask you to come to clinic in one year after your surgery to check on your wound for the last time if there is hernia or contacted by phone call in case you cannot come to the clinic. The charges for this extra clinic visit will be covered by the research money. You will also be compensated for transportation and parking at this clinic visit. During this visit, a member of the research team will administer the Quality of Life (QOL) questionnaire. You can abstain from answering questions that you do not feel comfortable with. The duration of the questionnaire will be of 10 minutes. The total duration of the visit will be of 30 minutes.

**2) Risks as a result of participating in the study:**

We expect that the new closure technique will result in lower risk of having wound complications than the classic closure technique. In addition, there may be unforeseeable risks from the new closure technique that we are not currently aware of.

**3) Benefits as a result of participating in the study**

If you participate in this study, you will help surgeon understand if the new technique has better results than the old technique, and thus choose the better technique for future patients.

**4) Any alternative treatment**

There is no other alternative to closing the abdominal wall other than the regular technique that we now use and the new technique that we will perform in this research study.

If you agree to participate in this research study, the information will be kept confidential. Unless required by law, only the study doctor and designee, the ethics committee and inspectors from governmental agencies will have direct access to your medical records

**5) Withdrawal from study after recruitment**

Your decision to enter the study is voluntary and will not affect your care during the surgery and hospitalization. You can withdraw from the study at any point with no consequences to your care. If you wish to withdraw at any point please contact the principal investigator Dr. Sbaity at 76/110882 or by email [es25@aub.edu.lb](mailto:es25@aub.edu.lb), or you can contact the study coordinator Ms. Rana Farsakouri at 71-203841. The principal investigator may exclude you from the study at any time if you no longer meet the inclusion criteria.

**6)** AUBMC will cover the cost of treating, on its premises, medical adverse events resulting directly from the medication and/or procedures of this research study. Otherwise, it will not cover for the costs of medical care for any medical condition or issue. .

**7) Any significant finding in this study will be conveyed to you if you are interested. If you want us to contact you at the conclusion of the study, please provide us with your information.**

**8) You will not be paid for taking part in this study.**

**PART II: Consent**

**Investigator’s Statement:**

**I have reviewed, in detail, the informed consent document for this research study with (name of patient, legal representative, or parent/guardian) the purpose of the study and its risks and benefits. I have answered to all the patient’s questions clearly. I will inform the participant in case of any changes to the research study.**

**_______________________**

**Name of Investigator or designee Signature**

**Date & Time**

**Patient’s Participation:**

**I have read and understood all aspects of the research study and all my questions have been answered. I understand that refusal to participate will not result in loss of benefits of treatment at the AUB-MC. I voluntarily agree to be a part of this research study and I know that I can contact Dr. Eman Sbaity at 76/110882 or any of his/her designee involved in the study in case of any questions. If I feel that my questions have not been answered, I can contact the Institutional Review Board for human rights at 01-350000 ext 5445. I understand that I am free to withdraw this consent and discontinue participation in this project at any time, even after signing this form, and it will not affect my care or benefits. I understand that the principal investigator may decide to take me off the trial for any reason he considers significant. I know that I will receive a copy of this signed informed consent.**

**__________________________**

**Name of Patient or Legal Representative Signature**

**or Parent/Guardian**

**Date & Time**

**Witness’s Name Witness’s Signature**

**(If patient, representative or parent does not read)**

**Date & Time**
