# Supplementary material for: Protocol for a randomized controlled trial comparing wound COmplications in elective midline laparotomies after FAscia Closure using two different Techniques Of Running sutures: COFACTOR trial
Source: Trials. 2020 Jul 2;21:608. doi: 10.1186/s13063-020-04507-8 (PMC7330541; doi:10.1186/s13063-020-04507-8)
Supplement: Supplementary file 2 — Additional file 2. Appendices. [file 13063_2020_4507_MOESM2_ESM.docx]

| Criteria For Defining A Surgical Site Infection (SSI)* | |
| --- | --- |
| **Superficial Incisional SSI** | Infection occurs within 30 days after the operation *and* infection involves only skin or subcutaneous tissue of the incision *and* at least *one* of the following:   1. Purulent drainage, with or without laboratory confirmation, from the superficial incision. 2. Organisms isolated from an aseptically obtained culture of fluid or tissue from the superficial incision. 3. At least one of the following signs or symptoms of infection: pain or tenderness, localized swelling, redness, or heat *and*superficial incision is deliberately opened by surgeon, *unless* incision is culture-negative. 4. Diagnosis of superficial incisional SSI by the surgeon or attending physician.   Do *not* report the following conditions as SSI:   1. Stitch abscess (minimal inflammation and discharge confined to the points of suture penetration). 2. Infection of an episiotomy or newborn circumcision site. 3. Infected burn wound. 4. Incisional SSI that extends into the fascial and muscle layers (see deep incisional SSI).   *Note:* Specific criteria are used for identifying infected episiotomy and circumcision sites and burn wounds.[[433](http://www.cdc.gov/hicpac/SSI/ref-SSI.html#433)] |
| **Deep Incisional SSI** | Infection occurs within 30 days after the operation if no implant† is left in place or within 1 year if implant is in place and the infection appears to be related to the operation *and* infection involves deep soft tissues (e.g., fascial and muscle layers) of the incision *and*at least *one* of the following:   1. Purulent drainage from the deep incision but not from the organ/space component of the surgical site. 2. A deep incision spontaneously dehisces or is deliberately opened by a surgeon when the patient has at least one of the following signs or symptoms: fever (>38°C), localized pain, or tenderness, unless site is culture-negative. 3. An abscess or other evidence of infection involving the deep incision is found on direct examination, during reoperation, or by histopatholog ic or radiologic examination. 4. Diagnosis of a deep incisional SSI by a surgeon or attending physician.   *Notes:*   1. Report infection that involves both superficial and deep incision sites as deep incisional SSI. 2. Report an organ/space SSI that drains through the incision as a deep incisional SSI. |

From the CDC guidelines: Centers for Disease Control and Prevention

**Wound Classification:**

American College of Surgeons. ACS data collection, analysis, and reporting [Internet]. Chicago, IL: American College of Surgeons; c2013 [cited 2012 Aug 31]. Available from: http://site.acsnsqip.org/programspecifics/data-collectionanalysis-and-reporting/

**BMI Categorization:**

Refer to this page on the NIH website.

<http://www.nhlbi.nih.gov/health/educational/lose_wt/BMI/bmi_dis.htm>

| **BMI (kg/m^2^)** | **Obesity Class** |
| --- | --- |
| **Underweight** | < 18.5 |
| **Normal** | 18.5–24.9 |
| **Overweight** | 25.0–29.9 |
| **Obesity** | >30.0 |

**Pain Assessment Scale**

**0–10 Numeric Pain Rating Scale**

0 1 2 3 4 5 6 7 8 9 10

No Moderate Worst pain pain possible

pain
